# Synergetic Effect of Polyaniline and Graphene in Their Composite Supercapacitor Electrodes: Impact of Components and Parameters of Chemical Oxidative Polymerization

**DOI:** 10.3390/nano12152531

**Published:** 2022-07-23

**Authors:** Olena Okhay, Alexander Tkach

**Affiliations:** 1 TEMA—Centre for Mechanical Technology and Automation, Department of Mechanical Engineering, University of Aveiro, 3810-193 Aveiro, Portugal; 2 LASI—Intelligent Systems Associate Laboratory, 4800-058 Guimaraes, Portugal; 3 CICECO—Aveiro Institute of Materials, Department of Materials and Ceramic Engineering, University of Aveiro, 3810-193 Aveiro, Portugal

**Keywords:** polymer, graphene, reduced graphene oxide, PANI, composites, chemical oxidative polymerization, supercapacitors, electrodes, specific capacitance, cycling stability

## Abstract

The current development of clean and high efficiency energy sources such as solar or wind energy sources has to be supported by the design and fabrication of energy storage systems. Electrochemical capacitors (or supercapacitors (SCs)) are promising devices for energy storage thanks to their highly efficient power management and possible small size. However, in comparison to commercial batteries, SCs do not have very high energy densities that significantly limit their applications. The value of energy density directly depends on the capacitance of full SCs and their cell voltage. Thus, an increase of SCs electrode specific capacitance together with the use of the wide potential window electrolyte can result in high performance SCs. Conductive polymer polyaniline (PANI) as well as carbonaceous materials graphene (G) or reduced graphene oxide (RGO) have been widely studied for usage in electrodes of SCs. Although pristine PANI electrodes have shown low cycling stability and graphene sheets can have low specific capacitance due to agglomeration during their preparation without a spacer, their synergetic effect can lead to high electrochemical properties of G/PANI composites. This review points out the best results for G/PANI composite in comparison to that of pristine PANI or graphene (or RGO). Various factors, such as the ratio between graphene and PANI, oxidants, time, and the temperature of chemical oxidative polymerization, which have been determined to influence the morphology, capacitance, cycling stability, etc. of the composite electrode materials measured in three-electrode system are discussed. Consequently, we provide an in-depth summary on diverse promising approaches of significant breakthroughs in recent years and provide strategies to choose suitable electrodes based on PANI and graphene.

## 1. Introduction

The current energy demands of humankind are continuously growing due to the increase of the number of portable electronic devices and electric vehicles. New technologies for energy generation from light (as solar cells), heat (as thermoelectric generators), or mechanical stimuli (as eolic/wind power generators) have appeared. At the same time, effective energy storage devices are still needed. Light, thin, eco-friendly, and flexible energy storage systems are also essential for portable and flexible electronic devices needs. Thus, high-efficiency electrochemical energy-storage devices, particularly supercapacitors (SCs), are receiving intensive interest due to their high power density and rate performance as well as their long cycling life [1,2,3]. In addition, SCs have shown high specific capacitance, rapid charge-discharge processes, and low maintenance cost in comparison to batteries.

Based on the mechanism of energy storage, supercapacitors can be divided into two main categories: electric double-layer capacitors (EDLC) and pseudocapacitors [2,4]. In EDLC, where carbonaceous materials are often used [3,5,6], the charge storage process is performed at the electrode/electrolyte interface. Thus, traditional carbonaceous EDLCs that store charge via a physical ion adsorption/desorption process present rather poor energy density but a long cycling life. The most studied carbonaceous material now is 2D graphene (G) with high mechanical strength and large specific surface area (SSA) (2630 m^2^/g [7]) that makes it an ideal electrode material for energy storage devices. Although reduced graphene oxide (RGO) cannot show an ideal structure as graphene, it is significantly cheaper than graphene monolayers and can present low sheet resistance < 200 Ω/sq [8] in addition to good chemical and thermal stability. Similar to graphene, RGO is tested as an electrode material in supercapacitors due to its rather high specific capacitance ~250 F/g [9] and SSA ~1520 m^2^/g [10,11,12,13], although it should be noted that a high SSA does not guarantee the rising specific capacitance [14,15,16,17].

In pseudocapacitors, energy can be stored through faradaic reactions in conductive polymers or metal oxides used as electrode materials [2,4]. These fast surface redox reactions take place during the charge/discharge processes which usually results in higher charge stored in pseudocapacitors compared to EDLC in cost of low electronic conductivity and/or poor structure stability.

**Figure 1:**
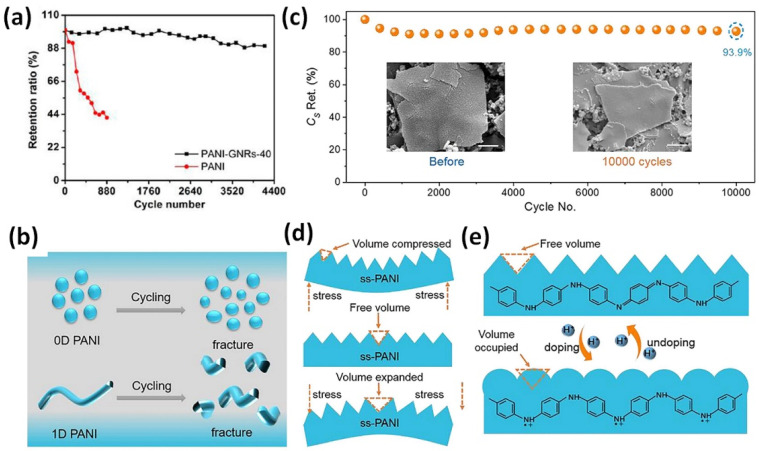
Cycling stability of pristine PANI and PANI with graphene nanorods (GNRs) (aniline to GNRs as 40:1) at a current density of 1 A/g measured in two-electrode cell (**a**) (Reprinted with permission from [30]. Copyright 2013 American Chemical Society). Illustration of structural disintegration of traditional conductive polymers that leads to poor cycling stability (**b**). Cycling stability of ss-PANI based flexible supercapacitor (**c**). The inset shows morphology unchanged before and after cycling. Illustrations of free volume between adjacent secondary mastoid structure can stabilized the ss-PANI through mitigating stress accumulation (**d**) and of doping/undoping processes within the structure that effectively mitigated the volume change (**e**). (Reprinted from [31], Copyright 2021, with permission from Elsevier).

As mentioned above, the electrode materials for supercapacitors mainly contain the carbon-based materials (e.g., carbon nanotubes (CNTs), activated carbon materials, and graphene) [18,19,20], transition metal oxides (RuO_2_, MnO_2_, TiO_2_, CoO_x_, V_2_O_5_ etc.) [21,22], and conducting polymers (polyaniline, polypyrrole, etc) [23,24]. In contrast to carbonaceous materials, charge storage in pseudocapacitive metal oxides is governed by Faradaic processes involving sub-valence state changes and enabling the development of high rate and energy density supercapacitors [25]. At the same time, metal oxides suffer from low electrical conductivity. Among the conducting polymers [26], polyaniline (PANI) is probably the most studied one due to its environment friendly nature, facile synthesis, and promising optical, electrical, and electrochemical properties [27,28,29]. However, poor electrochemical cycling stability of PANI (as can be seen in Figure 1a [30] and next subsections) restricts this low-cost supercapacitor electrode material from the viewpoint of commercial applications. PANI as the active material stores charge via redox reaction as the PANI transition between various oxidation states: fully reduced leucoemeraldine (LE), half oxidized emeraldine base (EB), and fully oxidized pernigraniline (PE). Both LE and PE are insulators in contrast to the intermediate PANI-EB that has high stability and conductivity [29]. However, the pseudocapacitive processes involve the swelling, shrinkage, and cracking of the polymer (as illustrated in Figure 1b) during doping/undoping of charged ions, resulting in poor cycling stability. In addition, the degradation of PANI may occur at relatively high potentials due to the over-oxidation, which lead to relatively low working potentials of the PANI electrode. These problems make it necessary to develop composite designs that couple other materials such as carbonaceous materials or metal oxides with the PANI matrix or to complicate the PANI preparation process. Very recently, Wang et al. employed a dynamically evolving emulsion polymerization strategy to elaborately construct two-dimensional supramolecular polyaniline nanosheets (ss-PANI) with significantly enhanced cycling stability (see Figure 1c) due to high volume durability and the intermolecular hydrogen bonding effect (Figure 1d), which efficiently restricted the volume change during repeatedly charging/discharging process (Figure 1e) [31].

From another side, G as well as RGO can show almost 100% cycling stability [3,5,6,32] and its combination with PANI results in more stable graphene/PANI composite electrode material after high number of charge/discharge cycles in the contrast to pristine PANI as also seen from Figure 1a. Thus, a great interest to a deep analysis of the synergetic effect within RGO and PANI composite is quite justified. Moreover, according to the major part of the available publications, PANI and graphene as well as PANI and RGO construct a composite by in situ electrochemical process or chemical oxidative polymerization that does not require any special equipment or additional precautions. In addition, the development and study of the composites made by the mix of RGO with already prepared PANI were also reported [33,34,35]. Furthermore, electrodes of PANI with graphene or RGO can be flexible and free-standing [35,36,37,38] that represents an advantage compared to conventional powdery electrodes, which usually need non-capacitive additives such as conductive agents/binder, leading to excessive contact resistance that is detrimental to the cycle performance of the electrode.

**Figure 2:**
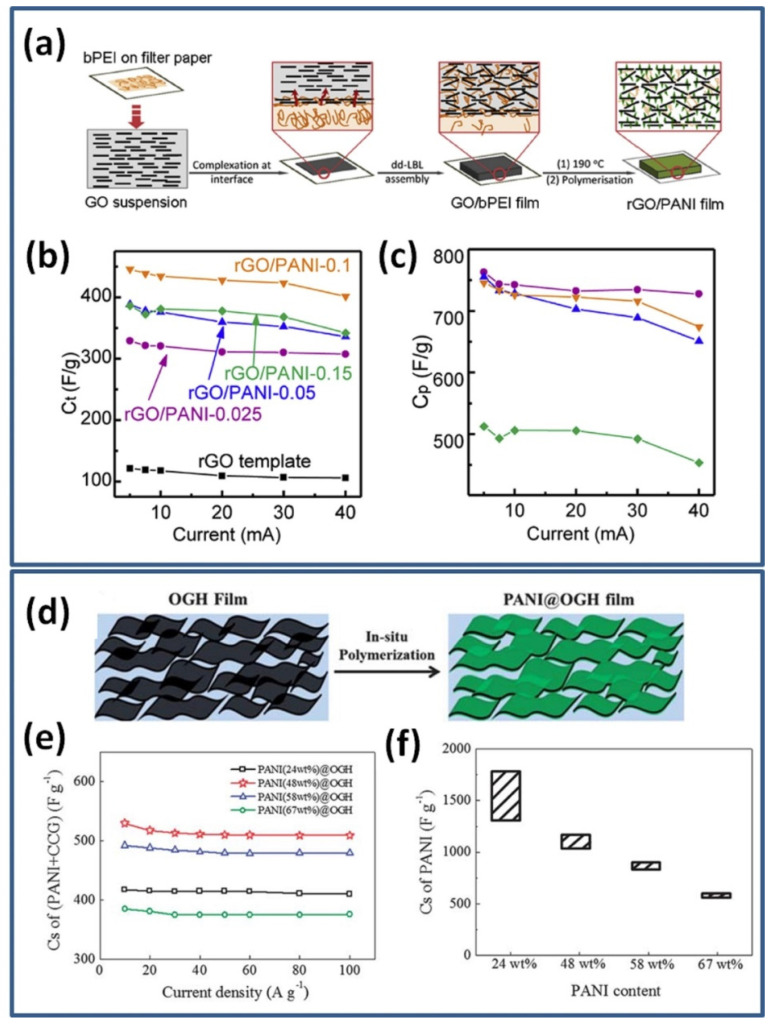
Schematic of preparing RGO/PANI composite film (**a**). Specific capacitances of RGO/PANI electrodes (**b**) and capacitance of PANI in RGO/PANI electrodes (**c**) at various charge/discharge currents (Reprinted from [39], Copyright 2017, with permission from Elsevier). Schematic of the PANI@OGH film formation (**d**). Specific capacitance of PANI@OGH films against the total weight of PANI and chemically converted graphene (CCG) (**e**), and specific capacitance of PANI against its own weight (**f**) at a current density of 10 A/g (Reprinted from [40] with permission of RSC Publishing).

Although graphene/PANI as well as RGO/PANI composites prepared by in-situ polymerization are widely studied as perspective electrode materials resulting in numerous publications, comparison, and deep analyses of the reported data is difficult due to high variation in electrode components, device configuration, thickness, mass loading, etc. Moreover, the reported capacitance of such composite electrodes can be shown as normalized against the weight of PANI only, when PANI is set as the only active material in composite, or PANI together with graphene or RGO as the active material (G/PANI or RGO/PANI, respectively). That makes the resulted energy and power densities also difficult to compare. A good example was shown by Hong et al. for an RGO/PANI electrode composite prepared using in-situ chemical oxidative polymerization method illustrated in Figure 2a [39].

The highest specific capacitance among used aniline concentrations from 0.025 to 0.15 M was calculated to be of 438 F/g based on the weight of both RGO and PANI (designated as C_t_ in Figure 2b). However, due to low capacitance of pristine RGO, Hong et al. decided to deduct the capacitance contributed by RGO from the whole capacitance and compared the remaining capacitances, which could be attributed to PANI (designated as C_p_ in Figure 2c). Obtained in this way specific capacitance increased significantly up to 763 F/g and did not show strong dependency on PANI content until very high values leading to structural changes such as aggregation [39]. 

A similar comparison can be seen for the filtered film of oriented graphene hydrogel (OGH) used for in-situ polymerization with different PANI contents (see Figure 2d) in work by Wang et al. [40]. The individual chemically converted graphene (CCG) sheets in the OGH film are largely separated by water, making it highly porous. In Figure 2e the specific capacitance calculated for full weight of electrode (RGO with PANI) is presented and shows the highest value of 530 F/g at 10 A/g for composite with 48 wt% PANI. However, Wang et al. mentioned that since the specific capacitance of analysed pristine OGH film was only 153 F/g, the contribution of graphene in the specific capacitance of composite can be neglected. Therefore, when specific capacitance was recalculated for PANI as the only active material the highest value of 1739 F/g was obtained for the composite with the lowest PANI content of 24 wt% (see Figure 2f). Thus, the reported specific capacitance data must be always clearly explained whether it is against the weight of PANI or against the weight of the whole active electrode (e.g., PANI with RGO) [40].

Based on the information above, the emphasis on this review will not be on showing the high capacitance values as well as values of high energy and power densities, which were calculated/presented in different ways, but on the routes of significant enhancement in the cycling stability and/or capacitance values of the carbonaceous material—PANI composite electrodes prepared using in-situ chemical oxidative polymerization in comparison to the pristine PANI or graphene-related material only. Also, there are just a few publications with graphene grown during the preparation process of the graphene-PANI composite, whereas the main part of researchers used graphene oxide (GO) as a starting material with the following transformation into RGO for the polymerization of PANI and preparation of the composites. Thus, when possible both graphene/PANI and RGO/PANI composites will be designated as G/PANI in the current review for the simplicity. 

## 2. Graphene/Polyaniline Composites as Supercapacitor Electrodes

The synthesis of PANI polymer from aromatic compounds on different substrates by chemical oxidative polymerization (COP) has been widely used and reported [41,42]. During the COP process, covalent bonds between monomer molecules are formed. In details, oxidation of aniline (ANI, C_6_H_5_NH_2_) monomers is achieved by using an oxidizing agent, such as ammonium persulfate (APS, (NH_4_)_2_S_2_O_8_). The polymer starts to grow when the monomer molecule is thus activated by an oxidant resulting in aniline molecule chain of higher molecular mass. The COP of G/PANI is a simple and fast procedure since G and ANI monomer just need to be mixed usually in acid solution (mainly HCL or H_2_SO_4_) with the following addition of APS as oxidant. At the same time, it is difficult to control precisely the morphology and component distribution of PANI-based materials during in-situ polymerization. For example, highly porous G hydrogel (GH) is an excellent conductive matrix for PANI, but in situ polymerization of aniline in this porous G matrix, either chemical or electro-chemical, yields a non-uniform PANI coating due to the competition between diffusion and polymerization in a porous substrate. PANI has a high solubility in several organic solvents, but there are very few wet methods for fabrication of PANI electrode, possibly due to the difficulty involved in producing a PANI nanostructure using the solution-assisted processing method (which is essential for high capacitive performance). 

### 2.1. Increasing Cycling Stability of Polyaniline

As it was mentioned above, pristine PANI electrodes typically have low cycling stability. Table 1 compiles the literature results and is shows that the cycling stability can be as low as 24% after 1000 cycles at 10 A/g [43]. Moreover, the cycling stability of pristine PANI was not reported to be higher than 70% in a major part of the publications except the reports on 87% (after 200 cycles at 1 A/g) [44] and 78.9% (after 1000 cycles at 10 A/g) [45]. However, the addition of graphene to electrolyte can increase the cycling stability of PANI for all studied G/PANI composites as presented in Table 1. 

Although modified graphene was used in the major part of the publications, there are several articles with unmodified RGO such as a work by Chen et al. with the cycling stability of G/PANI composite increased from 55% to 81.1% after 2000 cycles at 100 mV/s with RGO [61]. Moreover, 9 wt% GO used for preparation of G/PANI composite by hydrothermal (HT) method was reported as the optimal concentration compared to 2 and 3 wt%. Specific capacitance of the composite increased from 474 F/g for 2 wt% to 524 F/g for 9 wt% GO [61].

**Figure 3:**
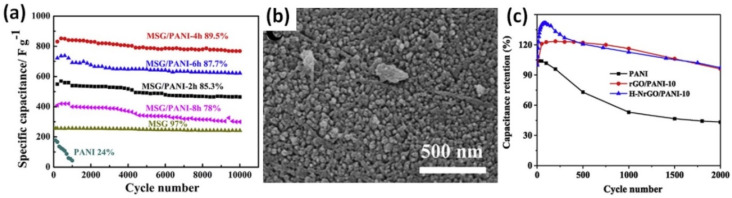
The cycling properties of samples at a current density of 10 A/g (**a**) and microstructure patterns of MSG/PANI after 4 h of polymerization (**b**) (Reprinted from [43], Copyright 2018, with permission from Elsevier). Cycling stability of the PANI, RGO/PANI-10 and H-NRGO/PANI-10 electrodes by cyclic voltammetry at 100 mV/s in H_2_SO_4_ (**c**) (Reprinted from [59], Copyright 2019, with permission from Elsevier).

As it can be seen from Table 1, the highest enhancement of the cycling stability for PANI was reported by Zheng et al. after a use of ~32 wt% of 3D multi-growth site graphene (MSG) treated by HNO_3_, H_2_O_2_ for in-situ polymerization of graphene/PANI composite [43]. In this work, pristine PANI electrode being degraded after 1000 galvanostatic charge-discharge (GCD) cycles at 10 A/g has shown the cycling stability of 24% in contrast to 89.5% after 10,000 cycles calculated for the composite of MSG and PANI (see Figure 3a). Moreover, the stability as well as the capacitance was found to strongly depend on the time of the polymerization that will be discussed in detail in one of further sections. The composite material obtained after 4 h of polymerization presented PANI nano-arrays on the surface of graphene sheets (see Figure 3b) that led to increase of the specific capacitance from 280 F/g at 1 A/g for pristine PANI to 912 F/g for MSG/PANI [43]. 

Liu et al. prepared a hybrid composite by COP of aniline in the presence of the holey nitrogen (N)-doped graphene oxide (H-NGO) reduced with hydrazine [59]. The presence of 10 wt% of N-doped RGO in the composite led to a significant difference (more than 2.2 times) between the cycling stability of 43% after 2000 GCD cycles at 100 mV/s for pristine PANI and 97% for its composite with N-doped RGO (H-NRGO/PANI) as shown in Figure 3c. In addition, the specific capacitance was also increased from 347 F/g to 746 F/g at 1 A/g by the N-doped RGO [59]. Moreover, Liu et al. also reported that after 2000 cycles, many pores in cross-section of PANI became fluffy that was explained by the degradation of PANI, due to the hydrolysis in emeraldine/pernigraniline structure of PANI in aqueous electrolyte. In contrast, many small pores were possible to be observed in H-NRGO/PANI after 2000 cycles, implying a more stable structure of the prepared composite material. Thus, declining specific capacitance could be explained as a result of the aggregation of the electroactive materials due to the phase separation that reduce the electrical conductivity of the electrode [59].

**Figure 4:**
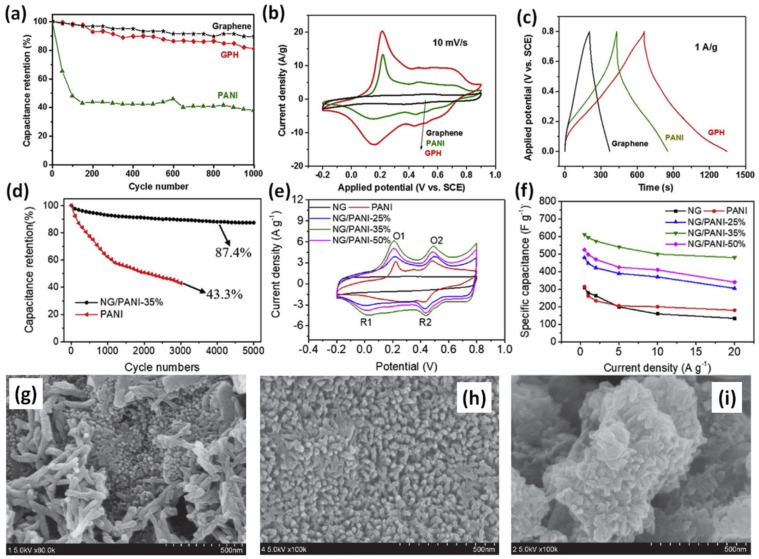
Cycling stabilities at 5 A/g (**a**), CV curves (**b**) and GCD curves (**c**) of graphene, PANI, and GPH at the current density of 1 A/g (Reprinted from [48], Copyright 2018, with permission from Elsevier). Capacitance retention of NG/PANI-35% and PANI electrodes after 5000 cycles at 5 A/g (**d**); CV curves of NG, pure PANI and NG/PANI electrodes with different percentage of PANI at 10 mV/s and 0.5 A/g, respectively (**e**); Specific capacitance of NG, pristine PANI and NG/PANI with different percentage of PANI at different current densities (**f**) (Reprinted from [65], Copyright 2020, with permission from Elsevier). Scanning electron microscopy (SEM) images of PAFG2 (**g**), PAFG5 (**h**) and PAFG10 (**i**) composite PANI with 2, 5 and 10 wt% amino-triazine functional reduced graphene oxide, respectively (Reprinted from [57], Copyright 2014, with permission from Elsevier).

A graphene/PANI hydrogel (GPH) composite with 9 wt% graphene prepared by in-situ COP of aniline with the aid of phytic acid (PA) had cycling stability as high as 82% after 1000 GCD cycles at 5 A/g increasing by 44% from 38% reported for pristine PANI (see Figure 4a) [48]. Moreover, as can be seen in Figure 4b the specific capacitance was increased in comparison to pristine PANI (from 531 F/g to 856 F/g at 1 A/g) while the duration of charge/discharge process was more than doubled (see Figure 4c). Moreover, PA was mentioned to be used as a protonation agent for doping PANI and as a physical cross-linker forming 3D hydrogel architecture [48].

Over 44% growth of cycling stability from 43.3% after 3000 cycles for pristine PANI to 87.4% after 5000 cycles at 5 A/g for the composite with 65 wt% N-doped graphene (NG) prepared by HT method with ethylenediamine (EDA) (see Figure 4d) was reported by Ge et al. [65]. However, cyclic voltammetry (CV) measurements of the composites presented typical for PANI redox peaks and CV area increased with the NG content up to 35 wt% as shown in Figure 4e. The specific capacitance also doubled, from 310 F/g for pristine PANI to 620 F/g at 0.5 A/g for PANI with the addition of NG, as seen in Figure 4f. Moreover, NG/PANI with 25 wt% and 50 wt% NG showed lower specific capacitance of 525 F/g and 480 F/g, respectively, at 0.5 A/g while the capacitance of NG was 317 F/g [65].

Ke et al. reported that only 5 wt% of amino-triazine (AT) functional reduced graphene oxide (ATRGO) were enough to change the cycling stability from 47% after 1500 cycles at 100 mV/s for pristine PANI to 89% for ATRGO/PANI [58]. Moreover, the specific capacitance more than tripled from 487 F/g at 1 A/g for pristine PANI) to 1510 F/g for the composite. In addition, lower (2 wt%) and higher (10 wt%) concentrations of ATRGO led to decreasing specific capacitances [58].

With a similar small concentration, only 5 wt% of amino-functionalized graphene (AFG) (modified by p-Phenylenediamine (p-PDA)) was reported as the optimal one for increase of the cycling stability from 47% after 1500 charge-discharge cycles at 100 mV/s for pristine PANI to 88% for composite of PANI with AFG (designated as PAFG). At the same time the specific capacitance of pristine PANI was also increased from 487 F/g to 1295 F/g after addition of AFG [57]. The optimized content of composite with 5 wt% amino-functionalized graphene exhibits homogeneous and dense PANI nanorods as shown in Figure 4h. Lower (2 wt%, Figure 4g) or higher (10 wt%, Figure 4i) AFG concentrations could not achieve fully oriented arrays of PANI nanorods morphology, and randomly stacked PANI nanorods or lower ordered PANI nanorods arrays can be observed [57]. Yu et al. reported that 10 wt% of modified graphene can increase the surface area of PANI, calculated according to the theory of Brunauer, Emmett, and Teller (BET), from 48 m^2^/g to 58 m^2^/g [62]. Moreover, cycling stability of the composite consisted of PANI with 10 wt% tetrabutylammonium hydroxide (TBAH) stabilized microwave-exfoliated graphene (MEG) sheets (TMEG) was reported to be 90% after 2000 cycles at 100 mV/s that is much higher than 56% reported for pristine PANI. Correspondingly, the specific capacitance grew from 764 F/g (PANI with 2 wt% TMEG) to 1255 F/g (PANI with 10 wt% TMEG) which is much higher than the capacitance of pristine PANI (626 F/g) [62].

Moreover, the specific capacitance value of ~1225 F/g reported by Liu et al. for the composite of PANI and similar 10 wt% graphene modified by sulfonated triazine was ~2.5 times higher than that of ~487 F/g reported for pristine PANI [56]. The composite cycling stability of 85.7% after 1500 cycles at 100 mV/s was also obtained against that of 47% for PANI [56].

**Figure 5:**
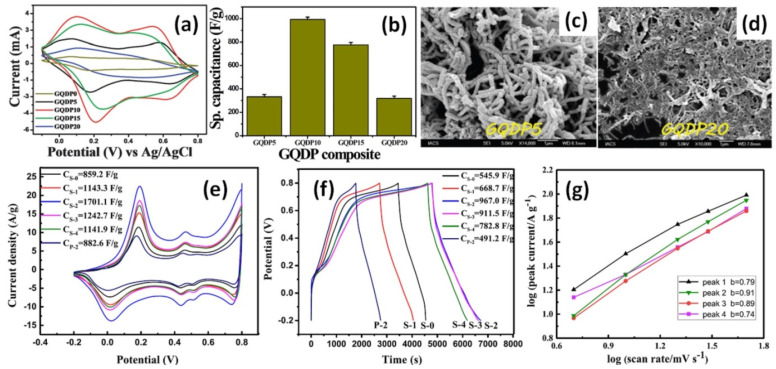
CV study of GQDP composites at the scan rate of 100 mV/s (**a**), Specific capacitance value of different GQDP composites (**b**), SEM images of synthesized GQDP composites having 5 wt% (**c**) and 20 wt% (**d**) GQDs (Reprinted from [70] with permission of RSC Publishing). CV curves of G-PC electrodes at a scan rate of 5 mV/s (**e**), GCD curves of G-PC electrodes at current density of 0.5 A/g (**f**) (0, 10, 20, 30, 50 wt% GNS-NH_2_ designated as S-0, S-1, S-2, S-3, S-4), and logarithm peak current *vs* logarithm scan rate plots of S-2 (inset: a summary of the calculated *parameter b* values) (**g**) (Reprinted from [66], Copyright 2020, with permission from Elsevier)].

Graphene quantum dots (GQDs)—PANI nanofiber composites (GQDP) with different GQDs contents were prepared by Mondal et al. using the chemical oxidation of aniline. The highest cycling stability of 80.1% after 3000 cycles at 1 A/g was obtained for composites with 10 wt% GQDs (see Figure 5a,b) [70]. Maximum specific capacitance of 1044 F/g was also obtained for GQDs/PANI composite electrode with 10 wt% GQDs in contrast to 206 F/g for pristine PANI. The diameter of PANI nanofibers was reported to increase with the GQDs content up to 15 wt% and decrease with the following addition of GQDs. At the same time, the nanofiber structure dominated in the composite with lower GQDs content (see Figure 5c) providing better conductive paths for fast electron transport in the contrast to the composites with high GQDs concentration (see Figure 5d) [70].

Li et al. studied in-situ polymerized PANI with 4-aminophenyl modified graphene (GNS-NH_2_) that presented cycling stability increase up to 56.5% after 4000 cycles at 500 mV/s in comparison to 35% for pristine PANI [66]. The highest specific capacitance of 1701 F/g at 5 mV/s (see Figure 5e) or 967 F/g at 0.5 A/g (see Figure 5f) was calculated for composite with 20 wt% GNS-NH_2_ (designated as S-2) that is much higher than 859 or 546 F/g, respectively, reported for pristine PANI (designated as S-0 in Figure 5e,f). Moreover, in contrast to mentioned above G/PANI composites, material analysed by Li et al. presented very strong oxidation peaks with GNS-NH_2_ concentration increase up to 20 wt% (see Figure 5e) [66]. To understand the energy storage mechanism the parameter *b* was deduced by Li et al. from the lope in *log(peak current) vs. log(scan rate)* plot based on CV results as shown in Figure 5g. According to the literature [2], the value of *parameter b* = 1 corresponds to the presence of the fast surface redox reaction and charge/discharge process inherent to EDLC when diffusion contribution is absent and CV shows linear current response dependency on the scan rate (*i~v*) as can be seen in Figure 5e for studied composite with 20 wt% GNS-NH_2_. At the same time, the peak current response of a battery-type electrode with strong redox peaks will be proportional to the square root of the scan rate (*i~v^1/2^*) and in this case *parameter b* = 0.5 [2,3]. The obtained by Li et al. *parameter b* for the four redox peaks vary from 0.74 to 0.91 (see Figure 5g), meaning that the kinetic in composite electrode with 20 wt% GNS-NH_2_ is mainly controlled by the pseudo-capacitive process (see Figure 5e) and the prepared composite is a promising candidate for the application in the energy storage devices.

Wang et al. studied the influence of RGO amino-functionalized by hexadecyl trimethylammonium bromide (CTAB) on the properties of RGO/PANI electrodes. The high cycling stability of 85% was obtained for such composites, although just for 800 cycles at 1 A/g, in contrast to 51% only for pristine PANI [47]. 

The tripled cycling stability from 29% after 3000 cycles at 100 mV/s to 87.6% was reported by Chen et al. after PANI polymerization with RGO modified by 4-methylaniline [64]. The specific capacitance of the obtained composite slightly increased to 530 F/g in comparison to 368 F/g reported for pristine PANI [64]. 

An example of a very long life electrode was reported by Hoa et al. for the three-dimensional PANI grafted with reduced graphene oxide (RGO-g-PANI) composite, showing 91.3% stability after 3000 cycles at 4 A/g and 1600 F/g specific capacitance at high current density of 12 A/g [71]. For that, an aerogel was prepared from graphene oxide grafted PANI (GO-g-PANI) composite obtained with N-Hydroxysuccinimide (N-HSM) and N-(3-(dimethylamino)propyl)- N’-ethylcarbodiimide hydrochloride (N-DNE) by hydrothermal and drying processes. The porous structure of the aerogel can facilitate electrolyte ion trapping and access to the surface of the electrode, which will enhance the electrolyte ion transportation during the charge/discharge process [71]. 

Thus, based on the analysis of Table 1 and discussion above one can summarize that there is no strong relationship between the content of graphene-related materials used in G/PANI composites and values of cycling stabilities as well as the specific capacitance of the analysed composite electrodes. However, all composite electrodes are always more stable for numerous charge/discharge cycles and have higher values of the gravimetric capacitance than pristine PANI. 

### 2.2. Rising Specific Capacitance of Graphene-Related Material 

Besides the reports on the cycling stability enhancement in G/PANI composites in comparison to the values mentioned for PANI (summarized in Table 1), there is a number of articles without the data for PANI but with the results for the pristine graphene-related materials and G/PANI composites (see Table 2). In these articles, the comparison is mainly carried out between the specific capacitance of graphene-related materials without and with PANI polymerization, since the cycling stability of pristine graphene is obviously/always higher than that of PANI.

As one can see in Table 2 (as well as in Table 1), the two- to four-fold increasing specific capacitance of graphene-materials can be observed in the composites with different PANI contents. However, several works reported atypical up to 16-fold increase for the composites of PANI with 10 wt% TMEG [62] or with 80 wt% mixed GO with pristine graphene (PG) [77]. At the same time, both these graphene-related materials showed relatively low initial values of the specific capacitance of 115 F/g and 50 F/g for TMEG and GO/PG, respectively. Typically, the specific capacitance ranges from 90 F/g at 5 mV/s [78] to 303 F/g at 0.5 A/g [49] for unmodified graphene-related materials without PANI and from 88.9 F/g at 1 A/g [79] to 347 F/g at 1 A/g [58] for the modified one, according to Table 2. 

Regarding the G/PANI composites, several groups reported very high specific capacitance of 1510 F/g at 1 A/g [58] or 1295 F/g at 1 A/g [57] for those containing as much as 95 wt% PANI or 1225 F/g at 1 A/g [56,62] for those with 90 wt% PANI, which were also found to be very stable, being thus described in the previous sub-section. At the same time, a strong increase of the specific capacitance from 316 F/g at 1 mV/s for RGO prepared with ethylene glycol to 1126 F/g for the composite with PANI was possible to obtain with PANI content in G/PANI composite as low as 7.7 wt% [90]. 

**Figure 6:**
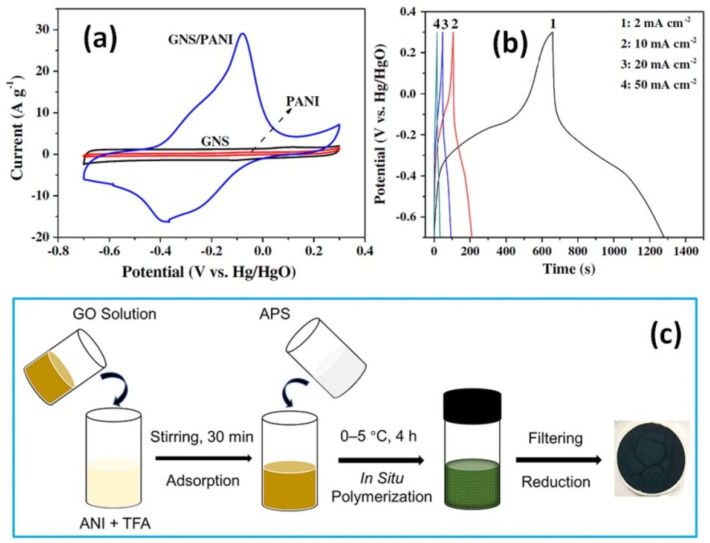
CV curves of GNS, pristine PANI and GNS/PANI composite at 10 mV/s (**a**) and GCD tests of GNS/PANI composite within the potential window −0.7 to 0.3 V (vs. Hg/HgO) at different current densities of 2, 10, 20 and 50 mA/cm^2^ (**b**) (Reprinted from [86], Copyright 2010, with permission from Elsevier). Schematic diagram of the synthesis of RGO/PANI composites (**c**) (Reprinted with permission from [85]. Copyright 2020 American Chemical Society).

Among the results described in Table 2, one of the highest variations in the specific capacitance was also reported by Yan et al., showing the values variation from 183 F/g at 1 mV/s for pristine RGO (called graphene nanosheets, GNS) to 1046 F/g for electrodes with 15 wt% RGO coated onto the nickel foam with addition of carbon black and poly(tetrafluoroethylene) (see Figure 6a) [86]. One should note however that GCD curves shown in Figure 6b as well as the calculated values of energy density (>100 Wh/kg) can belong rather to battery-type materials [2,3].

Freestanding RGO/PANI composites obtained by vacuum filtration after polymerization of the mixed solution of GO, trifluoroacetic acid (TFA), ANI, and APS (see Figure 6c), with GO/ANI ratios as 1:2 (33 wt% GO), 1:6 (14 wt% GO) with the following freeze-drying and chemical reduction were studied by Zhao et al. [85]. The highest specific capacitance was found to be 810 F/g at 1 A/g for the RGO/PANI electrode with 20 wt% GO [85]. Wang at al. have shown that the specific capacitance of unmodified graphene nanosheets measured without and with PANI has more than five-fold difference from 102 F/g to 532 F/g at 2 mV/s, respectively [80]. Moreover, the cycling stability of such GNS-PANI composite was still as high as 99.6% after 1000 cycles at 50 mV/s or 94% after 2000 cycles.

Thus, it is possible to summarize that there is no strong correlation between the increasing specific capacitance of graphene (or RGO) and the content of PANI in the composite probably due to large variation of other processing factors such as time, temperature, etc. for the PANI formation that will be discussed in the next subsections. However, one can clearly postulate that the addition of PANI to graphene significantly increases the final capacitance in the composite comparing to that of both pristine graphene-related material and PANI, while the cycling stability of composite with graphene is always higher than that of pristine PANI.

## 3. Optimised In-Situ Chemical Oxidative Polymerization Processing Details

As can be seen from the literature overview summarized in Table 1 and Table 2, the reported data of G/PANI composites can have a large variation from one to another case despite the same in-situ COP process of PANI. That should be related to different COP key parameters such as temperature of the polymerization, the process time, electrolyte components and their number, etc. Their variation can be crucial for the final electrode performance. For example, the polymerization of pristine PANI is well known to be possible in the wide temperature range from −20 to +40 °C [91]. However, an increasing content of structure defects in PANI chains was reported as the polymerization temperature is increased. A complex analysis of these results have shown that these defects do not consist of a partial self-doping of PANI chains by sulfonic or other acidic groups but are due to the presence of meanwhile unspecified structure irregularities. The content of structure defects is almost negligible in PANI prepared at 0 °C or −20 °C but significant for PANI prepared at the room or higher temperatures [91]. 

In the major part of the articles on G/PANI composites obtained by in-situ COP of aniline monomer, the used temperature of about 0–5 °C was reported and only in few works (i.e., by Kim et al. [46] or Salunkhe et al. [79]), the composites were prepared below 0 °C. Kim et al. mentioned −9 °C polymerization temperature since the anilinium hydrochloride used in that work became partially insoluble in the aqueous phase at this temperature and co-existed in both the aqueous and organic phase. In particular, phenyl group (the hydrophobic part of the anilinium hydrochloride) was adsorbed on the aromatic surface of RGO by the π–π interaction in the organic phase and the polymerization of aniline occurred on the surface of the RGO after addition of APS [46]. Moreover, Ates et al. demonstrated almost 30% difference between the specific capacitance of 324 F/g (2.78 F/cm^2^) and 245 F/g (1.75 F/cm^2^) measured in two-electrode cell for graphene hydrogel (GH) with PANI prepared at 0 °C and at +25 °C, respectively. Such difference was explained by dissimilar loading masses of 7.14 mg/cm^2^ for GH and GH/PANI at 0 °C, and 8.93 mg/cm^2^ for GH/PANI at 25 °C with the corresponding difference in the morphology. At the same time, the electrical conductivity of ∼136 S/m for the GH/PANI composite obtained at 25 °C was four orders of magnitude lower than 120 × 10^4^ S/m reported for GH/PANI prepared at 0 °C [92].

Thus, changing at least one of the processing details can result in significant variation of the properties of prepared materials as also described in the next subsections.

### 3.1. Aniline Monomer Content, Oxidants and Acids 

Sikdar et al. used different ratios between ANI (3.2 mmol) and APS (0.16, 8, 16 mmol) to prepare solutions in HCl before immersing RGO hydrogel (GH) there and the polymerization of PANI [93]. After controlled saturation process, the as-formed hybrids had different mass loadings from 3 mg/cm^2^ (PANI-GH1 with the lowest APS content) to 8.8 mg/cm^2^ (PANI−GH3 with the highest APS amount). All prepared electrodes exhibited EDLC behaviour with pseudocapacitive energy storage. Moreover, PANI-GH3, which contains the highest loading of PANI (83%) with respect to the weight of the hydrogel, thus presented the highest capacitance value of 503 F/g at a current density of 5 A/g followed by PANI-GH2 (80.7% PANI loading) and PANI−GH1 (50% PANI loading) [93]. 

**Figure 7:**
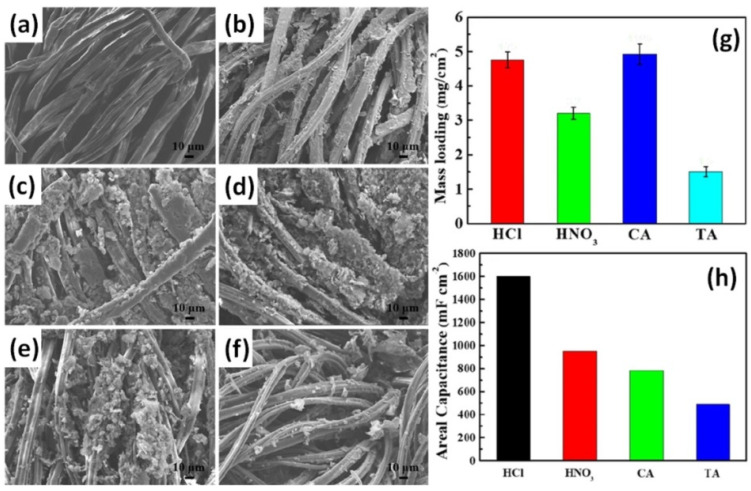
SEM images of the commercially available pristine textile (**a**), graphene on textile (**b**), PANI/G/T-HCl (**c**), PANI/G/T-HNO_3_ (**d**), PANI/G/T-CA (**e**) and PANI/G/T-TA (**f**). Mass loading of PANI with different acid dopants (**g**), the calculated areal specific capacitances four PANI/G/T-based electrodes (**h**) (Reprinted from [94], Copyright 2020, with permission from Elsevier).

The effect of the acidic dopants on the morphological, structural, and capacitive characteristics of the PANI/graphene/textile electrodes synthesized using a simple “dipping and drying” procedure followed by in-situ polymerization was investigated by Song et al. [94]. They used HCl, HNO_3_, D-tartaric acid (TA) or citric acid (CA) for the preparation of acid solutions that led to different structure of the final electrodes made on textile (T) covered by graphene (G/T) or PANI with graphene composite (PANI/G/T) (see Figure 7a–f) due to the dissimilar mass loading of PANI observed for different acids as shown in Figure 7g. Thus, PANI/graphene/textile electrode prepared with HCl (PANI/G/T-HCl) was found to depict a maximum areal specific capacitance of 1601 mF/cm^2^ at the current density of 1 mA/cm^2^ as seen in Figure 7h [94].

Double-crosslinked network functionalized graphene (by p-Phenylenediamine) and PANI (prepared with PA) stiff hydrogels (DN-PGH/PANI_PA_) have been successfully synthesized by polymerization of ANI in a confined functionalized graphene hydrogel (PGH) framework [95]. For comparison, single-network functionalized graphene/PANI hydrogels without acid and with H_2_SO_4_ (designated as SN-PGH/PANI_No-acid_ and SN-PGH/PANI_H2SO4_, respectively) were also prepared and analysed. The pore structure with predominant mesopores and minor macropores was identified for the materials and an average pore size of 11.7, 16.3 and 10.2 nm was calculated for DN-PGH/PANI_PA_, SN-PGH/PANI_H2SO4_ and SN-PGH/PANI_No-acid_, respectively. This porous structure can optimize the ion diffusion path and promote charge transport. The specific surface area of PGH is determined to be 18.2 m^2^/g. DN-PGH/PANI_PA_, SN-PGH/PANI_H2SO4_ and SN-PGH/PANI_No-acid_ possess a large specific surface area of 84.4, 74.4 and 108.2 m^2^/g, respectively. This result could be attributed to the incorporation of a large amount of PANI into the PGH framework that results in the superior areal specific capacitance of 3488 mF/cm^2^ reported for DN-PGH/PANI_PA_ [95]. 

**Figure 8:**
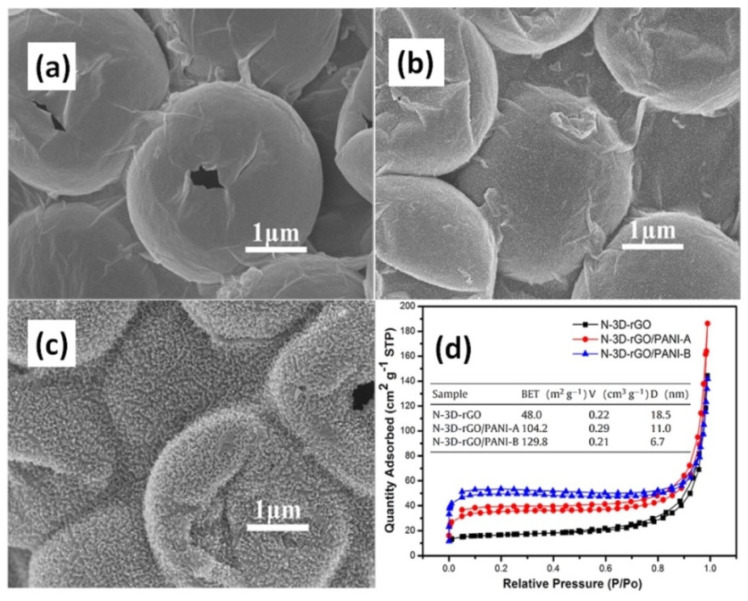
SEM images of N-3D-RGO (**a**), N-3D-RGO/PANI-A (**b**) (prepared with APS), N-3D-RGO/PANI-B (**c**) (prepared with β-MnO_2_), and their nitrogen adsorption/desorption isotherms (insert: BET surface area, pore volume (*V*), and pore diameter (*D*) (**d**) (Reprinted from [96], Copyright 2017, with permission from Elsevier).

In addition to different acids studied for the PANI electrolyte, commonly used APS as oxidizing agent was compared by Liu et al. with β-MnO_2_ during the polymerization process of N-doped RGO/PANI (N-RGO/PANI) electrodes [96]. For that 3D N-RGO powder was mixed with polystyrene nanospheres to be used as a template for the preparation of foam by hydrothermal and freeze-drying processes with addition of melamine (see Figure 8a). In-situ polymerization occurs by mixing 3D N-RGO, aniline monomer and APS (the final composite designated as 3D-RGO/PANI-A, Figure 8b) or β-MnO_2_ (designated as N-3D-RGO/PANI-B, Figure 8c) in HClO_4_. With N-3D-RGO as the supporting materials, the structure remains unchanged after PANI was loaded, thereby facilitating electrolyte penetration and rapid ion diffusion. N-3D-RGO/PANI polymerized with typical APS oxidant presented lower value of specific surface area of 104.2 m^2^/g in comparison to 129.8 m^2^/g for N-3D-RGO/PANI prepared with β-MnO_2_ (see Figure 8d). However, pore volume and pore diameter were larger when APS oxidant was used. The specific capacitance of prepared N-3D-RGO/PANI-A and N-3D-RGO/PANI-B were 187 F/g and 282 F/g at 1 A/g, respectively [96].

In summary, the specific capacitance can grow with increase of APS content due to higher polymer molecular mass and hence mass loading [93]. At the same time, the mass loading of active material can depend on used acid, being most enhanced by HCl, that results as well in high areal capacitance [95]. Moreover, β-MnO_2_ can be another promising oxidizing agent in addition to APS [96].

### 3.2. Polymerization Time

**Figure 9:**
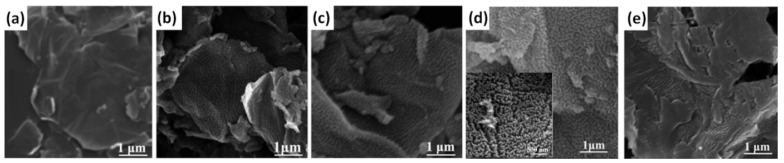
SEM images of PANI-RGO obtained at different reaction intervals: 1 h (**a**), 1.5 h (**b**), 2 h (**c**), 3 h (inset: high magnification SEM image) (**d**), 6 h (**e**) [55].

The polymerization in COP process starts immediately after the mixing of all components and the formation mechanism of the composites, and particularly the morphology, significantly depends on the process time. Figure 9 shows SEM images of PANI and aminophenyl-functionalized RGO composites polymerized during 1 h, 1.5 h, 2 h, 3 h and 6 h, respectively, reported by Wang et al. [55]. According to Figure 9a, a few short PANI nanowires grew on the surface of RGO after the reaction for 1.0 h. After 1.5 h, a large amount of PANI nanowires grew uniformly on RGO as seen in Figure 9b. The PANI nanowires gradually became longer as the reaction time increased (see Figure 9c,d). After 3 h, well-ordered PANI nanowire array was formed on RGO surface as shown by the high-magnification SEM in Figure 9d. Further increasing reaction time might lead to the aggregation of individual PANI and formation of compacted PANI nanowire films on RGO surface as seen in Figure 9e. Moreover, Wang et al. also mentioned that when suitable concentration of aniline was employed, homogeneous polymerization would not take place even at further increasing reaction time. The formation of compacted PANI nanowire films on RGO surface with further increasing reaction time might be ascribed to the high flexibility of PANI chains. It was very difficult for the longer PANI chains to stand vertically on RGO surface owing to its high flexibility. Accordingly, a bent structure was more favourable than a straight one as mentioned in previous works. With further increasing reaction time, the vertical PANI nanowires on RGO became longer and longer. The end of PANI chains stacked together to finally form compacted PANI nanowire films on RGO surface. Thus, 3 h was the optimal time in comparison to 1, 1.5, 2 and 6 h for such composite to obtain the specific capacitance of 590 F/g at 0.1 A/g [55].

Hou et al. studied and concluded that reaction time needed to yield the highest specific capacitance depends on ANI content put in the composite [63]. For the composites with high amount of PANI (ANI:RGO as 20:1 and 15:1), the specific capacitance was reported to decrease with the polymerization time. Composite with the higher ANI content in relation to RGO synthesized by using 1,2,4-triaminobenzene dihydrochloride (ANI:RGO as 20:1 that corresponds to ~95 wt% ANI and ~5 wt% RGO) presented the predominant PANI nanowire array morphology and just after 0.5 h polymerization revealed the highest specific capacitance of 597 F/g at 0.5 A/g. However, the cross-linking nanowires presented on the nanowire arrays of PANI disappeared at the ANI:RGO mass ratios lower than 10:1 (corresponds to ~83 wt% ANI and ~17 wt% RGO) and longer time of polymerization of 1 h was needed for the highest final capacitance of 527 F/g. Moreover, the specific capacitance monotonously increased from 370 F/g after 0.5 h to 398 F/g after 3 h polymerization time for composites with an ANI:RGO ratio of 5:1 [63].

**Figure 10:**
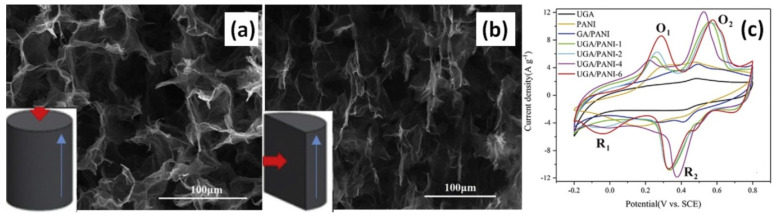
SEM images of UGA in top-view UGA/PANI (**a**), UGA/PANI in side-view (**b**) The blue arrow represents direction of ice crystal growth. CV curves of UGA, PANI, GA/PANI and UGA/PANI composites at a scan rate of 10 mV/s (**c**) (Reprinted from [52], Copyright 2018, with permission from Elsevier).

Much longer time from 3 to 12 h was studied by Bulin et al. for the polymerization of the immersed disk of p-PDA functionalized graphene aerogel into solution with ANI and APS [38]. The CV loop area increased with the reaction time up to 9 h and after that decreased [38]. At the same time, Wu et al. mentioned that just 4 h is the optimal polymerization time among 1, 2, 4, and 6 h for non-modified but prepared with the unidirectional structure graphene aerogel (UGA) (see Figure 10a,b), fabricated by unidirectional freeze casting of GO dispersions and high temperature reduction [52]. After polymerization during 4 h and implantation of UGA/PANI composite into graphite electrode the obtained composite subsequently displayed specific capacitance of 538 F/g at 1.0 A/g with cycling stability of 74% after 1000 cycles. However, when the polymerization time was increased to 6 h, the nanowires of PANI were thicker which might cause its underutilization for energy storage, inducing the decrease of the specific capacitance (see Figure 10c) [52]. 

The strong redox peaks were detected for multi-growth site graphene (MSG) with PANI composites soaked in HCl aqueous solution with aniline and APS during 2, 4 and 6 h. However, the peaks disappeared after 8 h of the process [43]. The load fraction of PANI in MSG/PANI was also reported to gradually increase with the reaction time as 41.32% for 2 h, 68.10% for 4 h, 76.83% for 6 h and 84.82% for 8 h. Moreover, the highest specific capacitance of 912 F/g at 1 A/g was obtained for MSG/PANI composite after 4 h polymerization time [43]. 

In addition, Kung et al. measured self-assembled PANI with ABF-G film with the highest capacitance of 643 F/g at 1 A/g obtained after 12 h of polymerization that corresponded to the high deposition loading and film thickness. However, CV area of the composites prepared during 18 and 24 h decreased [67].

Thus, polymerization time has a huge effect on the morphology of the obtained composite through formation of PANI structure as nanowire or nanowire arrays or nanobump arrays, etc. That structure can completely cover the conductive graphene-related materials leading to decrease of pore size as well as electrical conductivity that finally can decrease the specific capacitance of electrode at excessive PANI content.

## 4. Additives to Graphene/Polyaniline Composites 

### 4.1. Metal Oxides and Hydroxides

As a possible approach to further optimize the capacitive performances of the electrode materials, metal oxides or hydroxides were added as a third component into G/PANI composites [97,98,99,100,101,102,103,104,105]. A strong connection between metal oxide or hydroxide nanoparticles and 2D graphene resulted in unique structural features of composites and synergistic effect in the electrochemical properties derived from components of composite materials.

MnO_2_ is widely studied as a promising electrode material in general and for G/PANI-based supercapacitor electrodes in particular [97,98,99]. Usman et al. also used KMnO_4_ to obtain MnO_2_ with the addition of APS during the polymerization process of PANI with graphene grown on Ni foam (NFG) to prepare NFG/MnO_2_/PANI composite that demonstrated the specific capacitance of 815 F/g, while that for NFG/MnO_2_ composite was 650 F/g [97]. 

**Figure 11:**
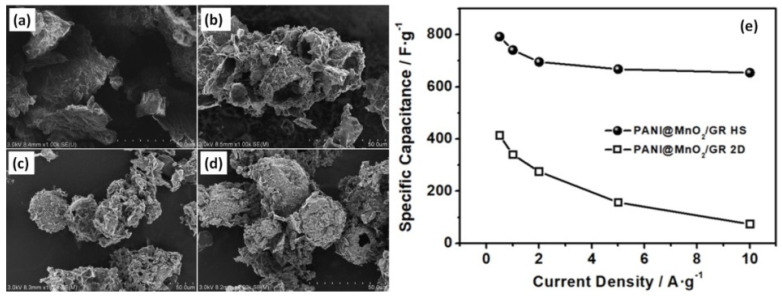
SEM images of PANI@MnO_2_/GR HS prepared with 0 (**a**), 2 (**b**), 4 (**c**), and 10% (**d**) of aniline content in the oil phase for the preparation of HS from KMnO_4_. Specific capacitance vs current density of PANI@MnO_2_/GR HS (prepared with 4% aniline to obtain HS) and PANI@MnO_2_/GR 2D (prepared by direct addition of aniline to the MnO_2_/GR aqueous dispersion) (**e**) (Reprinted with permission from [98]. Copyright 2021 American Chemical Society).

KMnO_4_ as well as manganese sulphate monohydrate (MnSO_4_×H_2_O) was used to obtain MnO_2_-modified graphene (MnO_2_/GR) composite electrodes [98]. The contact angle of MnO_2_/GR was found to strongly decrease with the KMnO_4_ amount: from 62.4 ± 0.4° (0.012 g of KMnO_4_ for 10% MnO_2_) to 13.8 ± 0.2° (0.436 g of KMnO_4_ for 80% MnO_2_). Moreover, Wang et al. used MnO_2_/GR as the water phase and toluene containing a certain volume fraction of aniline was used as the oil phase for the preparation of MnO_2_-modified graphene ternary hollow sphere (designated by MnO_2_/GR HS). Moreover, the amount of aniline in the oil phase was also noted as the main key for the formation process of the hollow sphere. Although no sphere could be observed without aniline loading (see Figure 11a) or with low aniline content of 2 % (Figure 11b), when the content was 4% (Figure 11c) and above, then definitive spherical formations were observed as seen for 10% aniline in Figure 11d. At the same time, the highest specific capacitance of 741 F/g at 1 A/g was obtained for the electrode with 60% MnO_2_ in MnO_2_/GR HS that is much higher than 414 F/g reported for the composite prepared by direct addition of aniline to the MnO_2_/GR aqueous dispersion that resulted in a two-dimensional structure of PANI@MnO_2_/GR 2D (see Figure 11e) [98]. 

RGO nanoplatelets/Fe_3_O_4_/PANI electrodes (with mass ratio equal to 1:3:4) studied by Charandabinezhad et al. exhibited 87% cycling stability after 1000 cycles at 1 A/g and specific capacitance of 610 F/g, which was much higher than 57 F/g for Fe_3_O_4_, 213 F/g for RGO/Fe_3_O_4_ and 383 F/g for PANI [100]. At the same time, Li et al. fabricated PANI together with halloysite nanotubes/graphene hybrid aerogel (HGA) from graphene modified by γ-methoxypropyltrimethoxysilane and Fe_3_O_4_ loaded onto halloysite nanotubes that helped the HGA/PANI composite to reach 81% cycling stability after 1000 cycles at 1 A/g and the specific capacitance of 577 F/g [106].

Similar to MnO_2_ and Fe_3_O_4_, Co_3_O_4_ was also used to enhance the electrochemical performance of G/PANI composite [101,102,103]. For example, Li et al. mixed GO/PANI nanosheets with the metal salts as the precursors of metal oxide followed by hydrothermal treatment [101]. During this process, PANI particles were formed on the graphene surface, while metal oxide nanoparticles grew simultaneously and were confined within the PANI nanostructures. Moreover, the particle size of PANI-Co_3_O_4_ increased to 6–10 nm when the ratio of graphene to aniline was 1:10, and it was 15 nm for the ratio of 1:20. The highest specific capacitance of 938 F/g was found for the composite with 90 wt% Co_3_O_4_ that was higher than 713 F/g for the composites with 75 wt% or 500 F/g for 95 wt% Co_3_O_4_ as well as than 126 F/g for G/PANI. The prominent performance of G/PANI/Co_3_O_4_ with 90 wt% Co_3_O_4_ was attributed to the optimal balance of the specific surface area, particle size, and Co_3_O_4_ content, indicating that PANI can partly contribute to the capacitance. Its main function, however, is to enhance the conductivity of Co_3_O_4_ and coupling effects between graphene and Co_3_O_4_ [101]. 

In addition to the metal oxides described above, several more oxides such as ZrO_2_ [107,108], NiO [109], WO_3_ [107], and V_2_O_5_ [107] were used for G/PANI electrode modification. G/PANI composite with V_2_O_5_ was shown by Haldar et al. to possess higher specific capacitance in comparison to G/PANI with ZrO_2_ or with WO_3_ [107]. At the same time, the cycling stability of G/WO_3_/PANI composite was reported to be ~95% after 3000 cycles, which was higher in comparison to the 92% and 84 % reported for the G/PANI composites with ZrO_2_ and V_2_O_5_ measured at 4 A/g, 3 A/g and 10 A/g, respectively [107].

More complicated structures were obtained by combination of G/PANI with ZnMn_2_O_4_ [110] and MnFe_2_O_4_ [111,112]. An RGO/ZnMn_2_O_4_/PANI hybrid showed up to 297.8 F/g at 0.2 A/g as was reported by Bao et al. [110]. Sankar et al. reported a significant increase of the specific capacitance from 32 F/g for pristine MnFe_2_O_4_ up to 262 F/g for MnFe_2_O_4_/graphene/PANI composite with 5 wt% graphene [111].

**Figure 12:**
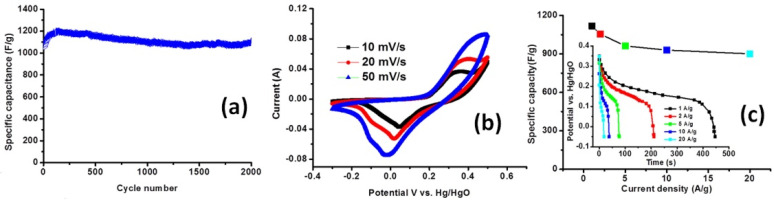
Cycle performance at the current density of 1 A/g (**a**), CV curves at various scan rates (**b**), and average specific capacitance versus discharge current density for GCD (**c**) of G/PANI/Ni(OH)_2_ (1:10). Inset shows GCD of G/PANI/Ni(OH)_2_ (1:10) at various discharge current densities (Reprinted with permission from [101]. Copyright 2013 Wiley).

Nickel hydroxide Ni(OH)_2_ was studied in G/PANI electrode [101] as well as cobalt hydroxide Co(OH)_2_ [104,105]. The specific capacitances above 1000 F/g for G/PANI/Ni(OH)_2_ (see Figure 12a) [101] and 2400 F/g for G/PANI simultaneously mixed with Co- and Ni-hydroxides [105] are the highest values among the reported ones. At the same time, such untypical for EDLC and pseudocapacitive materials high value of the specific capacitance, CV curve shape (see Figure 12b) and the visible plateau form of GCD curves (presented in inset of Figure 12c [101]) should rather correspond to the materials with the battery-type energy storage mechanism.

### 4.2. Metal Selenides and Nitrides

Also, a high value of specific capacitance was reported for G/PANI composites with zinc or molybdenum selenides. Thus, Xu et al. reported 1045 F/g at 1 A/g and 160% after 1000 cycles at 1 A/g for ZnS/RGO/PANI [113]. At the same time, a ZnS/RGO electrode prepared without PANI has shown much lower cycling stability of 88% after 1000 cycles at 1 A/g and as low specific capacitance as 425 F/g. 

MoS_2_ with G/PANI was studied by Chao et al. [114] and Li et al. [115]. MoS_2_/RGO@PANI retained over 82.5% of the starting value after 3000 cycles at 10 A/g and presented the capacitance of 1224 F/g that is higher than 774 F/g for PANI and 216 F/g for MoS_2_/RGO [115]. Slightly higher cycling stability of 95% after 5000 cycles at 5 A/g was reported by Chao et al. for P/MoS_2_/PANI/RGO hierarchical nanosheets with phosphorus (P) intercalated into the adjacent MoS_2_ layers enlarging the interlayer distance, although the specific capacitance of only 222 F/g was observed [114].

Graphitic carbon nitride (gC_3_N_4_) was studied by Mangisetti et al. for the application in energy storage device [116] similar to Maity et al., who reported the utilization of hexagonal boron nitride (BN) with various carbonaceous materials (GO, RGO, CNT) and PANI for supercapacitor application [117]. However, although BN/RGO/PANI has shown ∼101% stability after 1000 cycles, its capacitance of 326 F/g at 1 A/g was lower than 388 F/g for BN/CNT/PANI but higher than ~34 F/g reported for pristine BN or 74 F/g for BN/PANI or 192 F/g for BN/GO/PANI [117].

### 4.3. Carbon Nanotubes 

Besides the study of various carbonaceous materials in the composited with PANI, their combination was also investigated. A composite of graphene nanosheets, CNT, and PANI prepared by Sun et al. presented low specific capacitance of 18.3 F/g in the negative potential window (−2~−0.6 V) and much higher capacitance of 159 F/g in the positive potential part (−0.6~0.8 V) [118].

An even higher specific capacitance of 359 F/g at 1 A/g was obtained by Huang et al. for electrodes prepared by mixing RGO, CNT and aniline to obtain a composite with 80 wt% PANI and covering it onto glassy carbon electrode [119]. The composite showed CV with slight surface redox response from PANI and triangular CGD curves indicating the dominant capacitive behaviour in these electrodes [119]. 

**Figure 13:**
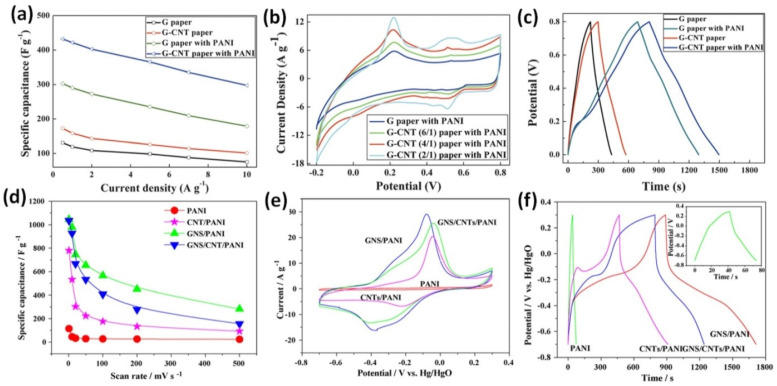
CV curves of G/PANI hybrid paper and G-CNT/PANI ternary hybrid papers at a scan rate of 10 mV/s (**a**); GCD curves of G paper and G–CNT composite paper with and without immobilized PANI particles at a current density of 0.5 A/g (**b**); specific capacitances of G paper and G-CNT composite paper with and without immobilized PANI particles as a function of current density (**c**) (Reprinted from [120] with permission of RSC Publishing). CV curves of pristine PANI, CNT/PANI, GNS/PANI, and GNS/CNT/PANI composite at 10 mV/s (**d**), GCD tests of pristine PANI and as-prepared composites at 2 mA/cm^2^ (**e**); specific capacitance of PANI and PANI-based composites at different scan rates (**f**) (Reprinted from [121], Copyright 2009, with permission from Elsevier).

Slightly higher 432 F/g at 0.5 A/g was observed for RGO/CNT/PANI freestanding paper obtained by filtration, which was much higher than 131 F/g for pristine RGO paper, 174 F/g for G-CNT composite paper, or 302 F/g reported for G/PANI hybrid paper (see Figure 13a) [120]. All analysed electrodes revealed pseudocapacitive behaviour as can be observed in Figure 13b showing CV curves and Figure 13c presenting CGD curves.

However, Yan et al. also studied the mixture of electrodes based on RGO, CNT, and aniline, and reported the capacitance of 1035 F/g at 1 mV/s as can be seen in Figure 13d (for the final composite including carbon black and poly(tetrafluoroethylene) (PANI content ~64 wt%)) [121]. However, the strong redox peaks shifting with the increasing scan rate in CV (see Figure 13e) as well as plateau in GCD curve (see Figure 13f) were observed [121], implying strong impact of battery-type energy storage. 

Thus, the addition of various metal oxides or hydroxides leads to an increase in specific capacity. However, with such an increase, there may be a change in the energy storage mechanism from the EDLC to the battery-type. So, the use of metal oxides and especially hydroxides should be carefully implemented and studied. At the same time, composites of graphene with PANI and with CNT additive present no battery-type but rather a strong capacitive/pseudocapacitive behaviour.

## 5. Symmetric Supercapacitors Based on PANI/RGO Electrodes

The energy density (in Wh/cm^3^) and power density (in W/cm^3^), as well as the cycling stability, are known to be among the main characteristic parameters of SC for their commercial application. Therefore, the goal of SC research is to achieve high energy density at high power density, although in the major part of the available publications these values are rarely presented (often being substituted by specific energy and power). SC specific energy (*E* in Wh/kg) and specific power (*P* in W/kg) can be calculated by using the following expressions:

(1) $$E=\frac{1}{2\times 3.6}C_{total}\Delta V^{2}\;\mathrm{or}\;E=\frac{1}{8\times 3.6}C_{single\;el.}\Delta V^{2},$$

(2) $$P=\frac{E}{\Delta t},$$

where *C_total_* and *C_single el._* are the measured capacitance of full SC and that of single electrode, respectively, ∆*V* is the operating voltage window, Δ*t* is the discharge time in hours. Thus, although the values of capacitance are very important for the SC performance, the electrolyte voltage window plays also a major role for the enhancement of specific energy as well as specific power.

In some articles mentioned above, two equal RGO/PANI electrodes were assembled to further investigate the electrochemical performance of symmetric supercapacitors. Summarizing the results, Table 3 includes the main characteristics for the reported symmetric capacitors made of G/PANI-based electrodes together with such parameters as the electrolyte kind and its potential window. The electrolyte can be solid such as H_2_SO_4_ with polyvinyl alcohol (PVA) or liquid such as H_2_SO_4_, Na_2_SO_4_ or tetraacetylammonium-tetrafluoroborate-acetonitrile (Et_4_NBF_4_-AN) with different potential windows up to 2 V. 

According to Table 3, the specific capacitance can be up to 700 F/g at 1 A/g [85]. Since the specific power of supercapacitors is typically higher than that in battery, the main interest here is in the value of the specific energy. As it can be also seen from Table 3, today the highest value of the specific energy can reach 62.2 Wh/kg for RGO/PANI [85] and 66.3 Wh/kg for GH/PANI [92]. The addition of CNT to RGO/PANI also leads to high specific energy up to 86.4 Wh/kg although it was measured in Et_4_NBF_4_-AN electrolyte with the highest potential window [118]. In all of these cases, high specific energy values beneficiate from EDLS and Faradaic contributions of RGO and PANI, respectively, while the cycling stability is above 90%. On the other hand, full supercapacitors based on G/PANI electrodes with rather moderate specific energy values can be very stable after a high number of cycling and can show almost no loss after 5000 cycles at 5 A/g [84] or even after 10,000 cycles at 5 [89] or 10 A/g [38].

## 6. Summary and Outlook

In the current review, recent advances in the synthesis, design and properties of graphene/PANI composites as electrode materials for supercapacitors were presented. Using different preparation conditions, ratios between components, methods, etc., the obtained composites show higher cycling stability than that of pristine PANI and higher specific capacitance than that of pristine graphene-related materials. However, a large variation of the different key factors such as time of the polymerization, types of oxidants, acids, and the ratio between the components can significantly change the morphology/mass loading/electrochemical performance of G/PANI composites. Therefore, the optimized synthesis conditions further enhance the cycling stability as well as the specific capacitive performances of G/PANI electrodes. 

In addition, no strong direct dependency was found between concentrations of PANI and graphene since their composite structure includes oxidizing agents such as APS and acids such as HCl or H_2_SO_4_. At the same time, the specific capacitance can be enhanced by the polymerization time optimisation that, in turn, strongly depends on the ratio between aniline and graphene. Also, an increase of APS content during the process can significantly increase mass loading as well as areal specific capacitance.

Moreover, we also stressed that due to the difference in the presentation view of the published results, the comparison of the specific capacitance values of G/PANI composites could be incorrect. At the same time, it is obvious that cycling stability of PANI always significantly increased with the addition of graphene and that the specific capacitance of graphene-related materials grew in composites with PANI. 

In addition, it is possible to propose the following topics for further investigations:

- To analyse the time (speed) of the charge/discharge process, which is the one of the main differences between the supercapacitors and batteries as well as between the shape of CV and GCD;
- To test the cycling stability for longer periods of time (more than 10,000 cycles), which is important for practical applications;
- To develop/improve new methods for preparation of the porous materials on an industrial scale.

Moreover, it must be understandable by the researchers that depending on the ratio in the composite between graphene and PANI, cycling stability will be more important for the composites with amount of PANI > 51 wt% (since its specific capacity is obviously higher than that for graphene); and the specific capacitance will be expected to increase if the concentration of graphene in the composite is higher than 51 wt% (since its cycling stability is already expected to be higher than that for pristine PANI). Thus, articles with comparative data between the initial materials and the final composites are undoubtedly valuable for scientists.

In addition, it is necessary to study and explain the charging mechanism of graphene/PANI composites as single electrodes before formation of the full device since the development and combination of capacitive or pseudocapacitive electrodes with the battery-type materials will result in hybrid device with perspective electrochemical performance in comparison to typical EDLC supercapacitors. It will also be important to study the graphene/PANI composite in various electrolytes, particularly in those for Na, K, and Mg-ion instead of Li-ion batteries.

In conclusion, graphene/PANI composites can be used as a basis for high-performance supercapacitors. However, more studies and tests in the future must be carried out in a systematic manner to obtain high-performance electrode materials and, respectively, supercapacitors for a wide range of applications.

## Figures and Tables

**Table 1:** Literature data of the cycling stability and specific capacitance measured in three-electrode systems for PANI and graphene-related components separately and in composites (ordered by number of cycles of cycling stability). Meaning of the abbreviations can be found in the text or glossary.

Materials for PANI-Based Composite	Used Graphene-Related Material (Starting Materials)	Amount of G in Composite, wt%	Cycling Stability of Pristine PANI//G/PANI Composite, % (at Number of Cycles/at Current Density or Scan Rate)	Specific Capacitance of PANI//G//G/PANI Composite, F/g (at Current Density or Scan Rate)	Ref.
HCl, ANI, APS	RGO (GO, HBr)	2	87//91 (200/1 A/g)	253//-//446	[44]
HCl, ANI, APS (ANI:APS as 1:1.2) CSA, m-cresol/ chloroform solution, AmS	RGO (GO, HydrM)	-	60//76 (500/0.45 A/g)	256//-//431 (5 mV/s)	[46]
HCl, ANI, APS (APS:ANI as 1:1)	RGO (NaBH_4_, NH_4_OH, CTAB)	-	51//85 (800/1 A/g)	298//113//421 (0.6 A/g)	[47]
HCl, ANI, APS	MSG (GO, ascorbic acid)	31.9	24 (1000)//89.5 (10,000/10 A/g)	280//253//912 (1 A/g)	[43]
PA, ANI, APS (ANI:PA:APS as 10:2:2.5)	RGO (GO, HydrH)	9	38//82 (1000/5 A/g)	531//209//856 (1 A/g)	[48]
HCl, ANI, APS (APS:ANI as 1:1)	RGO (GO, 160 °C) (ANI:GO as 1:20)	-	44//88 (1000/1 A/g)	311//303//648 (0.5 A/g)	[49]
HCl, ANI, APS	p-PDA-G (GO, NaBH_4_, p-PDA, NaNO_2_)	0.5	47//76 (1000/1 A/g)	380//138//440	[50]
PA, ANI, APS, p-PDA ((ANI and p-PDA):APS as 1:1) ((ANI and p-PDA):PA as 5:1)	RGO (GO, 180 °C) (GO:p-PDA:ANI as 1:1:25)	3.6	53//94.4 (1000/20 A/g)	448//-//538 (1 A/g)	[51]
HCl, ANI, APS	GA (GO, 1100 °C)	69	57//74 (1000/3 A/g)	312//243//538 (1 A/g)	[52]
HCl, ANI, APS	RGOA (GO, p-ABA, HCl, NaNO_2_, EtGl, AmS)	-	60.6//72.3 (1000 /10 A/g)	342//156//553 (1 A/g)	[53]
HCl, ANI, APS (ANI:APS as 2:1)	HQ-G (GO, HQ, 180 °C)	-	62//82 (1000/10 mA/cm^2^)	351//264 (1.14 A/g)//435 (22.73 A/g)	[54]
H_2_SO_4_, ANI, APS (ANI:APS as 4:1)	TBA-RGO (GO, HydrM, degassed acetonitrile, NDTF, TBAH, AcAc, Zn, NH_4_Cl)	-	69//81 (1000/2 A/g)	215//-//590 (0.1 A/g)	[55]
H_2_SO_4_, ANI, APS, SDS	GmH (GO, m-PDA), 180 °C) (PANI:GO as 7:1)	-	78.9//87.1 (1000/10 A/g)	325//-//514 (1 A/g)	[45]
H_2_SO_4_, ANI, APS	ST-GNS (GO, ST)	10	47//85.7 (1500/100 mV/s)	487//123//1225 (1 A/g)	[56]
HCl, ANI, APS	AFG (GO, HydrH, p-PDA, isoamyl nitrite)	5	47//88 (1500/100 mV/s)	487//238//1295 (1 A/g)	[57]
H_2_SO_4_, ANI, APS	AT-RGO (GO, HydrH, TCTA, p-PDA)	5	47//89 (1500/100 mV/s)	487//347//1510 (1 A/g)	[58]
HCl, ANI, APS	N-doped RGO (GO, NH_4_OH, H_2_O_2_, HydrH)	10	43//97 (2000/100 mV/s)	347//96//746 (1 A/g)	[59]
H_2_SO_4_, ANI, APS	GH (GO, 180 °C)	9	52//89 (2000/10 A/g)	401//291//618 (1 A/g)	[60]
HCl, ANI, APS (ANI:APS as 1:1)	RGO (GO, NaOH) (ANI:GO as 10:1)	9	55//81.1 (2000/100 mV/s)	397//-//524 (0.5 A/g)	[61]
H_2_SO_4_, ANI, APS	TMEG (MEG, TBAH)	10	56 (2000)//90 (2000/100 mV/s)	626//115//1225 (1 A/g)	[62]
HCl, ANI, APS (ANI:APS as 10:1)	TD-RGO (GO, TD, 180 °C)	20	65//89 (2000/1 A/g)	400//-//489	[63]
HCl, ANI, APS (ANI:APS as 1:1)	MA-RGO (GO, MA, 95 °C)	-	29//87.6 (3000/100 mV/s)	368//-//530 (0.5 A/g)	[64]
HClO_4_, ANI, APS (ANI:APS as 3:2)	N-doped G (GO, EDA, 180 °C)	35	43.3//87.4 (5000/5 A/g)	310//317//620 (0.5 A/g)	[65]
HCl, ANI, APS, p-PDA, TPA	GNS-NH_2_ (GO, HydrH, NaNO_2_, BD, H_2_SO_4_)	30	35//56.5 (4000/500 mV/s)	859//-//967 (0.5 A/g)	[66]
OSAN, ANI, APS	ABF-G (graphite powder, ABA, PPA, P_2_O_5_)	3	-	378//-//642 (1 A/g)	[67]
HCl, ANI, APS	RGO (GO, HydrH, 100 °C)	6	-//78.8 (1000/2 A/g)	318//-//496	[68]
H_2_SO_4_, ANI, APS (ANI:APS as 4:1)	G (GO, HydrH, 95 °C)	-	-//84 (1500/2 A/g)	333//-//596 (0.5 A/g)	[69]
Water, ANI, APS	GQDs (GO, H_2_O_2_, 90 °C)	10	-//80.1 (3000/1 A/g)	206//-//1044 (1 A/g)	[70]
HCl, ANI, APS, K_2_S_2_O_8_	N-grafted G (GO, ADF, N-HSM, N-DNE, DAP, 180 °C)	-	-//91.3 (3000/4 A/g)	600//-//1600 (12 A/g)	[71]
H_2_O, ANI, TSA, APS	GQDs (citric acid)	-	-//100 (7000/7 A/g)	93//-//245	[72]
HCl, ANI, APS	RGO (GO, HydrH)	2	-//-	323//-//552 (0.5 A/g)	[73]
HCl, ANI, APS	TBAOH-G (GO, TBAH, SDBS)	35	-//-	264//-//526 (0.2 A/g)	[74]
HCl, ANI, H_2_SO_4_, APS	S-N-doped GQDs (GO, citric acid, thiourea)	-	-//-	177//-//645 (0.5 A/g)	[75]
PA, HCl, ANI, APS	ABA-RGO (GO, NaBH_4_, NaNO_2_, ABA)	-	-//-	512//-//652	[76]

**Table 2:** Literature data for graphene-related component separately and in graphene/PANI composite measured in three-electrode systems (ordered by the values of the specific capacitance of reduced graphene oxide or graphene (G)). Meaning of the abbreviations can be found in the text or glossary.

Used Graphene-Material	Materials for PANI-Based Composite	PANI Amount in G/PANI Composite, wt%	Specific Capacitance of G//PANI//G/PANI, F/g (at Current Density or Scan Rate)	Cycling Stability of Composite, % (Cycle Number/at Current Density or Scan Rate)	Ref.
GO-PG (graphite powder, DMSO, Na_3_C_6_H_5_O_7_×2H_2_O)	HCl, ANI, APS	20	50//-//794 (1 A/g)	83.4 (1000/100 mV/s)	[77]
3D RGO (GO, NaCO_3_, CaCl_2_, CaCO_3_, glucose, NH_4_OH, 180 °C)	HClO_4_, ANI, APS	-	88.9//-//243 (1 A/g)	87 (1000/1 A/g)	[79]
RGO (GO, HydrH)	HClO_4_, ANI, APS	-	90//78//286 (5 mV/s)	94 (2000/50 mV/s)	[78]
N-doped RGO (GO, AmS, H_2_O_2_)	HCl, ANI, APS	90	96//347//746 (1 A/g)	97 (2000/100 mV/s)	[59]
GNS (GO, 180 °C)	HCl, ANI, APS	-	102//353//286 (2 mV/s)	94 (2000/50 mV/s)	[80]
3D-RGO (GO, CaCl_2_, AmS)	HClO_4_, ANI, APS	-	110//-//385 (0.5 A/g)	90 (5000/5 A/g)	[81]
RGO (NaBH_4_, NH_4_OH, CTAB)	HCl, ANI, APS (APS:ANI as 1:1)	-	113//298//421 (0.6 A/g)	85 (800/1 A/g)	[47]
TMEG (MEG, TBAH)	H_2_SO_4_, ANI, APS	90	115//626//1225 (1 A/g)	90 (2000/100 mV/s)	[62]
RGO (NaBH_4_, 95 °C)	HCl, ANI, APS	-	120//105//147 (0.5 A/g)	-	[82]
ST-GNS (GO, ST)	H_2_SO_4_, ANI, APS	90	123//487//1225 (1 A/g)	85.7 (1500/100 mV/s)	[56]
G (GO, HydrH)	HClO_4_, ANI, APS, chloroform	-	125//245//578 (1 A/g)	-	[83]
N,S-doped GH (GO, urea, triourea, 180 °C)	HCl, ANI, APS	-	130//-//237 (0.5 A/g)	95 (1000/10 A/g)	[36]
p-PDA-G (GO, NaBH_4_, P-PDA, NaNO_2_)	HCl, ANI, APS	99.5	138//380//440 (1 A/g)	76 (1000/1 A/g)	[50]
B-doped G (GO, H_3_BO_3_, 180 °C) (ANI:B-doped G as 1:1)	HCl, ANI, APS (ANI:APS as 1:1)	50	158//284//406 (1 mV/s)	90 (5000/2 A/g)	[84]
RGOA (GO, ABA, HCl, NaNO_2_, EtGl, AmS)	HCl, ANI, APS	-	156//342//553 (1 A/g)	72.3 (1000/10 A/g)	[53]
RGO (GO, HydrH)	H_2_O, TFA, ANI, APS (TFA:ANI:APS as 1:2:2)	80	156//325//810 (1 A/g)	-	[85]
GNS (GO, HydrH)	HCl, ANI, APS	85	183//115//1046 (1 mV/s)	-	[86]
RGO (GO, 180 °C) (GO: p-PDA:ANI as 1:1:25)	PA, ANI, APS, p-PDA (ANI + p-PDA):APS as 1:1 (ANI + p-PDA):PA as 5:1	-	190//-//610 (1 A/g)	94.4 (1000/20 A/g)	[51]
3D RGO (GO, HydrM, AmS)	HClO_4_, ANI, APS (ANI:APS as 1.5:1)	-	190//-//740 (0.5 A/g)	87 (1000/10 A/g)	[37]
3D G (HNO_3_, H_2_SO_4_, Ni NPs as template, 900 °C, Ar, H_2_, CH_4_)	H_2_SO_4_, ANI, APS (ANI:APS as 4:1)	-	201//-//680 (1 A/g)	76 (1000/10 A/g)	[87]
G (GO, HydrM, 95 °C)	HCl, ANI, APS	20	206//420//480 (1 A/g)	-	[88]
RGO (GO, sodium ascorbate, 95 °C)	HCl, ANI, APS, methylbenzene (ANI:APS as 4:1)	30	208//-//777 (1 A/g)	85 (6000/5 A/g)	[89]
RGO (GO, HydrH)	PA, ANI, APS (ANI:PA:APS as 10:2:2.5)	91	209//531//856 (1 A/g)	82 (1000/5 A/g)	[48]
p-PDA-AFG (GO, HydrH, p-PDA, isoamyl nitrite)	HCl, ANI, APS	95	238//487//1295 (1 A/g)	88 (1500/100 mV/)	[57]
GA (GO, 1100 °C)	HCl, ANI, APS	31	243//312//538 (1 A/g)	74 (1000/3 A/g)	[52]
MSG (GO, ascorbic acid)	HCl, ANI, APS	68.1	253//280//912 (1 A/g)	89.5 (10,000/10 A/g)	[43]
HQ-G (GO, HQ, 180 °C)	HCl, ANI, APS (ANI:APS as 2:1)	-	264 (1.14 A/g)//351//435 (22.73 A/g)	82 (1000/10 mA/cm^2^)	[54]
GH (GO, 180 °C)	H_2_SO_4_, ANI, APS	91	291//401//618 (1 A/g)	89 (2000/10 A/g)	[60]
RGO (GO, 160 °C) (ANI:GO as 1:20)	HCl, ANI, APS (APS:ANI as 1:1)	-	303//311//648 (0.5 A/g)	88 (1000/1 A/g)	[49]
RGO (GO, EtGl, NaOH, 90 °C)	HCl, ANI, APS (ANI:APS as 1:1)	7.7	316//777//1126 (1 mV/s)	84 (1000/0.2 A/g)	[90]
N-doped G (GO, EDA, 180 °C)	HClO_4_, ANI, APS (ANI:APS as 3:2)	65	317//310//620 (0.5 A/g)	87.4 (5000/5 A/g)	[65]
GA (GO, 140 °C, p-PDA)	HCl, ANI, APS	79.1	338//-//810 (1 A/g)	83.2 (10,000/-)	[38]
AT-RGO (GO, HydrH, TCTA, p-PDA)	H_2_SO_4_, ANI, APS	95	347//487//1510 (1 A/g)	89 (1500/100 mV/)	[58]

**Table 3:** Literature data of the symmetric supercapacitors made of PANI and graphene-related components (ordered by the electrolyte type).

Composite Electrodes	Electrolyte (Potential Window, V)	Specific Capacitance of Symmetric SC, F/g (at Current Density)	Specific Energy (Wh/kg)	Specific Power (W/kg)	Cycling Stability of Symmetric SC, % (at Number of Cycles/at Current Density or Scan Rate)	Ref.
MSG/PANI	H_2_SO_4_-PVA (0–+0.8)	120 (1 A/g)	30	850	90 (5000/10 A/g)	[43]
RGO/PANI	H_2_SO_4_-PVA (−0.2–+0.8)	700 (1 A/g)	62.2	800	91.3 (2000/5 A/g)	[85]
GO-PG/PANI	H_2_SO_4_ (0–+0.7)	564 (2 A/g)	50.2	2143.8	80 (1000/100 mV/s)	[77]
3D-RGO/PANI	H_2_SO_4_ (0–+0.7)	385 (0.5 A/g)	-	-	88 (5000/5 A/g)	[81]
GH/PANI	H_2_SO_4_ (0–+0.8)	503 (5 A/g)	29.85	1160	95.8 (3000/5 A/g)	[93]
ABA-RGO/PANI	H_2_SO_4_ (0–+0.8)	512 (1 A/g)	-	-	>100 (4000/5 A/g)	[76]
B-doped G/PANI	H_2_SO_4_ (−0.2–+0.6)	241 (0.5 A/g)	19.9	523.5	~100 (5000/5 A/g)	[84]
N-doped RGO/PANI	H_2_SO_4_ (−0.2–+0.8)	510 (1 A/g)	24.7	329.5	74 (2000/3 A/g)	[59]
3D G/PANI	H_2_SO_4_ (−0.2–+0.8)	72 (1 A/g)	6.43	400	78 (1000/10 A/g)	[87]
RGO/PANI	H_2_SO_4_ (−0.2–+0.8)	665 (1 A/g)	10.9	-	100 (10,000/5 A/g)	[89]
GA/PANI	H_2_SO_4_ (−0.2–+0.8)	211 (-)	~30	<50	~100 (10,000/10 A/g)	[38]
GH/PANI	H_2_SO_4_ (−0.2–+0.8)	311 (0.4 A/g)	66.3	539.9	99 (1000/100 mV/s)	[92]
GNS-NH_2_/PANI	H_2_SO_4_ (−0.2–+0.8)	110 (0.1 A/g)	15.3	50	94.9 (5000/500 mV/s)	[66]
RGO/PANI/	H_2_SO_4_ (−0.2–+0.8)	-	30	216	91.21 (1000/20 mV/s)	[73]
S-N-doped GQDs/PANI	H_2_SO_4_ (0–+1)	124 (1 A/g)	17.25	500	90 (1000/2.5 A/g)	[75]
RGO/PANI	H_2_SO_4_ (0–+1.6)	53 (2 A/g)	19.02	1599	94 (2000/50 mV/s)	[78]
RGO/MoS_2_/PANI	H_2_SO_4_ (0–+1)	160 (1 A/g)	22.3	5080	-	[115]
RGO/UCNTs/PANI	H_2_SO_4_ (0–+1)	53 (0.5 A/g)	7.4	189	-	[119]
3D PC-g/PANI	Na_2_SO_4_ (0–+1)	440 (2 A/g)	61	1000	94 (10,000/5 A/g)	[116]
*a*MWCNT/GNS/PANI	Et_4_NBF_4_-AN (−0.6–+2)	-	86.4	730	93 (10,000/-)	[118]

## Data Availability

No new data were created or analyzed in this study. Data sharing is not applicable to this article.

## Glossary

ABA	4-aminobenzonic acid
ABF-G	4-aminobenzoic acid- functionalized graphene
AcAc	acetic acid
AFG	amino-functionalized graphene
AmS	ammonia solution
ADF	anhydrous dimethylformamide
ANI	aniline monomer
*a*MWCNT	acid-treated multi-walled carbon nanotube
APS	ammonium peroxydisulphate
AT	amino-triazine
BA	benzoic acid
BD	1,4-benzenediamine
BET	Brunauer, Emmett, and Teller
CA	citric acid
CCG	chemically converted graphene
CNT	carbon nanotube
COP	chemical oxidative polymerization
CSA	camphorsulfonic acid
CTAB	hexadecyltrimethylammonium bromide
CV	cyclic voltammetry
DAP	1,3- diaminopropane
DMSO	dimethyl sulfoxide
EB	emeraldine base
EDA	ethylenediamine
EDLC	electric double-layer capacitors
EG	electrochemically exfoliated graphene
EtGl	ethylene glycol
Et_4_NBF_4_-AN	tetraacetylammonium-tetrafluoroborate-acetonitrile
G	graphene
GA	graphene aerogel
GF	graphene foam
GH	graphene hydrogel
GmH	hydrogel of graphene modified by m-phenylenediamine
GNS	graphene nanosheets
GNS-NH_2_	4-aminophenyl modified graphene
GNRs	graphene nanorods
GO	graphene oxide
GPH	graphene polyaniline hydrogel
GQDs	graphene quantum dots
HS	hollow sphere
HT	hydrothermal method
HQ	hydroquinone
HydrM	hydrazine monohydrate N_2_H_4_
HydrH	hydrazine hydrate (N_2_H_4_∙H_2_O)
IN	isoamyl nitrite
LE	leucoemeraldine
MA	4-methylaniline
MEG	microwave-exfoliated graphene sheets
MnO_2_/GR	MnO_2_-modified graphene
m-PDA	m-phenylenediamine
MSG	multi-growth site graphene
MW	microwave
N-DNE	N-(3-(dimethylamino)propyl)-N’-ethylcarbodiimide hydrochloride
NDTF	nitrophenyl diazonium tetrafluoroborate
NFG	graphene grown on Ni foam
N-HSM	N-Hydroxysuccinimide
OGH	oriented graphene hydrogel
OSAN	o-aminobenzenesuphonic acid
PA	phytic acid
PANI	polyaniline
p-ABA	p-aminobenzonic acid
PC-g	porous carbon-graphene
PE	pernigraniline
PG	pristine graphene
PGH	polyaniline graphene hydrogel
PPA	polyphosphoric acid
p-PDA	p-phenylenediamine
PVA	polyvinyl alcohol
RGO or rGO	reduced graphene oxide
RGOA	reduced graphene oxide aerogel
SC	supercapacitor
SDBS	sodium dodecylbenzene sulfonate
SDS	sodium dodecyl sulfate
SEM	scanning electron microscopy
SSA	specific surface area
ST	2,4,6-tri(40-aminobenzenesulfonic acid)-1,3,5-triazine
TA	D-tartaric acid
TBA	tetrabutylammonium
TBAH	tetrabutylammonium hydroxide
TCTA	2,4,6-trichloro- triazine
TD	1,2,4-triaminobenzene dihydrochloride
TFA	trifluoroacetic acid
TMEG	tetrabutylammonium hydroxide stabilized microwave-exfoliated graphene
TPA	triphenylamine
TSA	*p*—toluenesulfonic acid
UCNTs	unzipped carbon nanotubes
UGA	unidirectional graphene aerogel
